# Comparative analysis of postoperative pain after transvaginal hybrid NOTES versus traditional laparoscopic cholecystectomy in obese patients

**DOI:** 10.1007/s00464-021-08855-7

**Published:** 2021-11-03

**Authors:** Dirk R. Bulian, Sebastian Walper, Dana C. Richards, Sissy-A. Schulz, Claudia S. Seefeldt, Panagiotis Thomaidis, Jurgen Meyer-Zillekens, Markus M. Heiss

**Affiliations:** Department of Abdominal, Tumor, Transplant and Vascular Surgery, Cologne-Merheim Medical Center, Witten/Herdecke University, Ostmerheimer Strasse 200, 51109 Cologne, Germany

**Keywords:** Transvaginal NOTES, Cholecystectomy, Pain, Outcome, Obesity, Body mass index

## Abstract

**Purpose:**

Even though obesity is a known risk factor for needing cholecystectomy, most research excludes patients with higher degrees of obesity. The aim of this retrospective study was to compare postoperative pain and analgesic consumption in obese patients, who underwent either transvaginal hybrid Natural Orifice Transluminal Endoscopic Surgery (NOTES) cholecystectomy (NC) or traditional laparoscopic cholecystectomy (LC).

**Methods:**

Between 12/2008 and 01/2017, 237 NC were performed, of which 35 (14.8%) showed a body mass index (BMI) of 35 kg/m^2^ or more (obesity II and III according to the World Health Organization). Of these, procedural time, postoperative pain, analgesic requirements, and other early postoperative parameters were collected and compared with 35 matched LC patients from the same time period.

**Results:**

There were no differences in the baseline characteristics between the two groups, but we found significant benefits for the hybrid NOTES technique in terms of less pain (*P* = 0.006), coherent with significantly less intake of peripheral (paracetamol; *P* = 0.005), and of centrally acting analgesics (piritramide; *P* = 0.047) within the first two-day post-surgery. We also found that those in the NC group had shorter hospital stays (*P* < 0.001). The postoperative complication rates and the procedural time did not differ between the two groups.

**Conclusion:**

With regard to postoperative pain and analgesic requirements and without an increase in postoperative complications, obese patients experience short-term benefits from the hybrid NOTES technique compared to traditional laparoscopic cholecystectomy.

Transvaginal hybrid NOTES cholecystectomy with rigid instruments (NC), first performed by Zornig et al. in June 2007, has become established in some hospitals as an alternative to traditional laparoscopic cholecystectomy (LC) [1]. A meta-analysis of 13 studies demonstrated that, compared to LC, NC reduced postoperative pain and postoperative analgesic requirements, while accelerating postoperative convalescence and improving the esthetic surgical outcome without increasing intraoperative or postoperative complication rates [2, 3]. However, most studies excluded patients with higher degrees of obesity [4], which is a risk factor for cholecystolithiasis and hence the need for cholecystectomy [5]. In a 2018 comparative analysis, we demonstrated the feasibility of NC in patients with a particularly high body mass index (BMI), showing that although these patients required a longer operative time and had a significantly higher probability of additional trocar use, they experienced similar postoperative lengths of stay and complication rates when compared to patients with normal weight [4]. Thus, our question now was whether the advantages of NC also apply to higher-risk obese patients. Therefore, we compared short-term outcomes for obese patients with a BMI of 35 kg/m^2^ or more, on whom we operated using the NC technique, with obese patients who had an LC operation.

## Materials and methods

### Patients

**Fig. 1:**
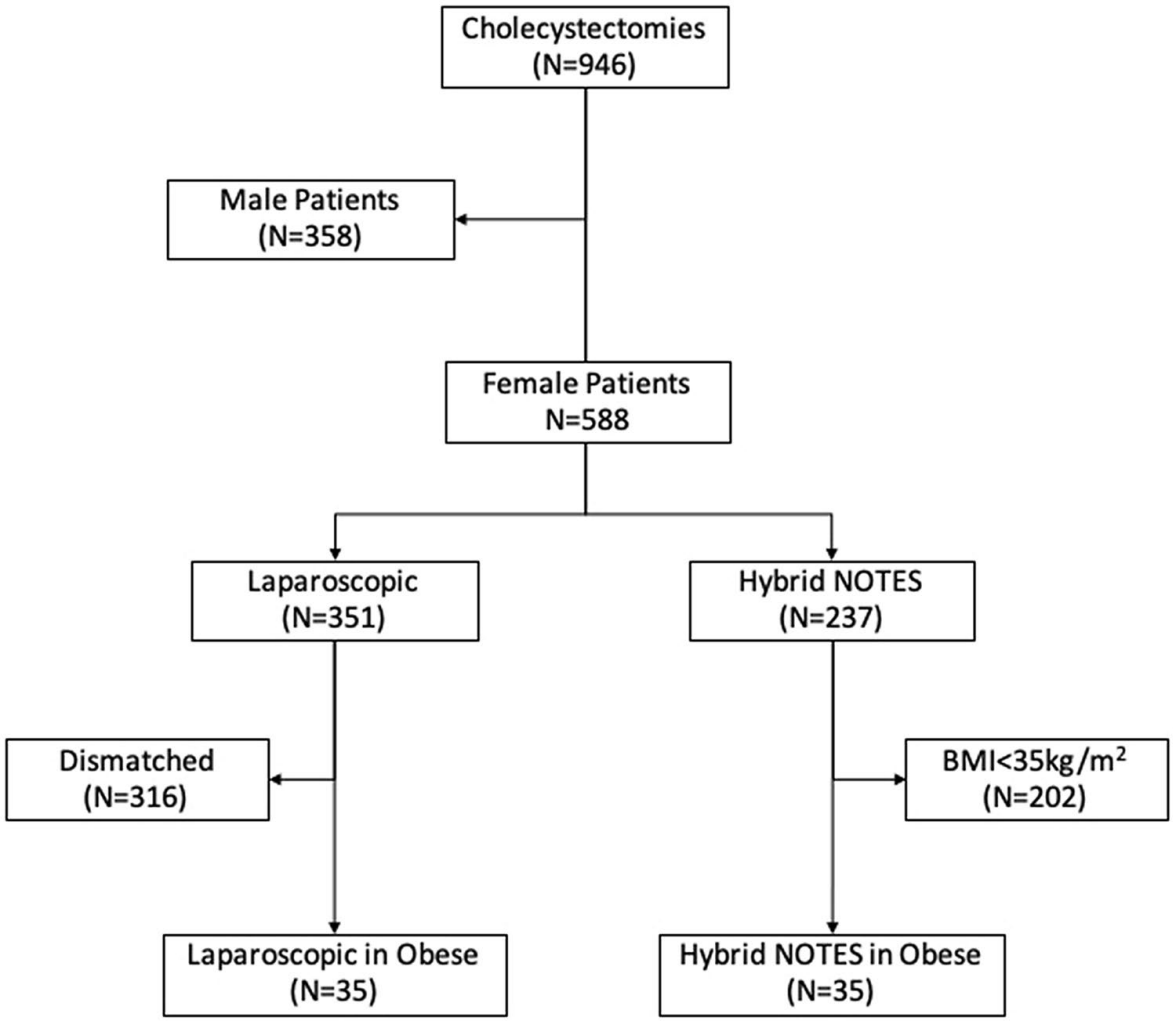
Trial flow diagram

Between December 2008 and January 2017, 946 cholecystectomies were performed at Cologne-Merheim Medical Center. Of these, 588 procedures were performed on female patients; 237 were performed using the hybrid NOTES technique and 351 using LC. Thirty-five of the 237 NC patients had a BMI of 35 kg/m^2^ or more, corresponding to obesity II° or III° according to the WHO classification. As a control group, the first 35 patients who matched the NC group in terms of BMI (≥ 35 kg/m^2^), age, and surgical urgency were selected from the 351 LC patients (Fig. 1). Furthermore, we assessed the ASA (American Society of Anesthesiologists) classification and history of major abdominal surgery. Notably, emergency surgery was excluded to obtain as homogeneous a comparison group as possible. Also, procedures differing from the standard technique (4-trocar technique with 6 mm as well as 11-mm trocars) were excluded when creating the traditionally laparoscopic control group.

### Inclusion and exclusion criteria for transvaginal approach

In principle, our clinic offers the hybrid NOTES technique to all female patients as an alternative to traditional laparoscopic surgery. Contraindications for the transvaginal procedure were as follows: nonadjustable cervix in patients who were not hysterectomized; ongoing pregnancy; genital infections; known endometriosis; and neoplasms of the vulva, vagina, or cervix. These criteria did not result in the exclusion of any patients from the TVC procedure.

### Surgical technique

Transvaginal/transumbilical hybrid NOTES cholecystectomy was performed with rigid reusable instruments in the lithotomy position. The surgeon stood on the left side of the patient with the assistant between the legs. The capnoperitoneum up to 11–15 mmHg was created via Veres needle, which was inserted at the umbilicus after a small incision in the depth of the navel. A 6-mm trocar (Karl Storz GmbH & Co KG, Tuttlingen, Germany) was inserted transumbilically after safety tests and removal of the Veres needle. A diagnostic laparoscopy was performed by a transumbilical 5-mm optic (45°, 29 cm long, Karl Storz GmbH & Co KG, Tuttlingen, Germany) and the Douglas was exposed in a Trendelenburg position. A curved 5-mm grasping forceps (according to CUSCHIERI O-CON, 43 cm long, Karl Storz GmbH & Co KG, Tuttlingen, Germany) and a transvaginal 11-mm trocar without connector for insufflation (Karl Storz GmbH & Co KG, Tuttlingen, Germany) were inserted via the posterior vault of the vagina under sight via the umbilical trocar. The gallbladder was hold with the curved grasper and the camera was changed to a 10-mm optic (45 degrees, 42 cm long, Karl Storz GmbH & Co KG, Tuttlingen, Germany), which was inserted via the transvaginal 11-mm trocar. The dissection of the gallbladder, the cystic duct, and the cystic artery, as well as clipping (Endo Clip 5-mm clip applier, Covidien, MA, USA) and transecting of them was performed via the umbilical 6-mm trocar in the standard fashion. The gallbladder was then transvaginally removed through the 11-mm trocar incision in the posterior vault using a retrieval bag (Endo Catch Gold, Covidien, MA, USA) after changing the view to the transumbilical 5-mm optic. The 2 small incisions in the posterior vault were closed with resorbable sutures. In difficult cases, an additional 6-mm trocar (Karl Storz GmbH & Co KG, Tuttlingen, Germany) was used at the right costal margin. The described technique was not changed throughout the period.

Traditional laparoscopic cholecystectomy was performed in the supine position with legs apart. The surgeon stood on either the left side of the patient with the assistant between the legs or vice versa. Four trocars (6 mm and 11 mm; Karl Storz GmbH & Co KG, Tuttlingen, Germany) were used and the capnoperitoneum up to 11–15 mmHg was created either via Veres needle or via the first 11-mm trocar, which was inserted at the umbilicus in an open technique. Dissection of the gallbladder did not differ between the two techniques and was performed regularly with the stromal hook. The cystic duct and cystic artery were closed centrally and peripherally with clips in each case after reaching the "view of safety." In all cases, the gallbladder was removed transumbilically in a retrieval bag (ExBag, Medi-Globe GmbH, Achenmuehle, Germany). The umbilical fascia and skin incision were widened according to the size of the gallstones and closed with sutures in each case.

Although the German guideline does not suggest the use of perioperative antibiotic prophylaxis in the context of elective laparoscopic cholecystectomy in low-risk patients, patients with obesity, among others, are considered high-risk patients, so that according to our intern guidelines all patients in this study received a preoperative, intravenous antibiotic single-shot prophylaxis with Ampicillin 2 g and Sulbactam 1 g [6].

At our hospital, there is no difference in the postoperative treatment after the techniques described. Full mobilization as well as oral feeding, in absence of nausea or vomiting, is intended on the day of surgery. The postoperative analgesic standard consists of 1000 mg paracetamol administered intravenously every 6 h on the day of surgery, 1000 mg paracetamol administered three times per os on the first postoperative day, and 500 mg paracetamol administered three times per os on the second postoperative day. In addition, the intravenous administration of 3.75 mg to 15 mg of piritramide (an opioid with a morphine-equivalence factor of 0.7) was provided if needed. Any standard pain medication not taken by the patients was documented by the nursing staff. If an allergy was known or occurred in the course, medication was adjusted accordingly. Thromboembolism prophylaxis consisted of a low-molecular weight heparin. Patients were discharged on the second postoperative day if clinical condition, wound healing, and blood test results allowed it. A discharge prior to the second postoperative day is financially penalized by the German health insurance billing system and thus not sought.

### Outcome parameter

In all patients, age, sex, height, weight, surgical technique, reason for cholecystectomy (symptomatic cholecystolithiasis; cholecystitis; choledocholithiasis; biliary pancreatitis), procedural time, analgesic administration (peripheral acting analgesics and centrally acting analgesics; regular and additional requirement), pain intensity, postoperative complications, and postoperative length of stay were documented. For NC, some of the parameters were prospectively entered into a dedicated registry and the remaining data were collected retrospectively from clinical documentation.

To be able to detect a difference in existing preoperative pain as well as to account for preoperative analgesic intake due to different underlying diseases, these were also documented and analyzed.

For postoperative pain assessment, the numeric rating scale (NRS) is used by trained nurses twice per day and additionally six hours after surgery, documenting the pain level between 0 (no pain) and 10 (worst imaginable pain).

Postoperative complications were classified and compared according to Clavien/Dindo [7].

### Statistics

The data were prepared in Microsoft Excel, and SPSS Statistics 27 (IBM Corp., Armonk, NY, USA) was used for the statistical analyses and data processing of all variables. Data of continuous variables are expressed as median and interquartile range. Binary and categorical variables are reported as counts and percentages. The Mann–Whitney U test was used for continuous parameters, the Chi-square test for categorical parameters, and the Chi-square test for trend for ordinally scaled variables. A *P*-value less than 0.05 was considered statistically significant.

## Results

The patient parameters such as age, height, weight, BMI, indication for cholecystectomy, ASA classification, history of major abdominal surgery, preoperative pain, and need for preoperative analgesics were all comparable and did not differ significantly between groups (Table 1).

**Table 1:** Baseline characteristics of all patients

Variable	NC (*n* = 35)	LC (*n* = 35)	Total (*n* = 70)	*P* value
Age [years]	43.0 (25–63)	45.0 (25–72)	43.5 (25–72)	0.182
Height [cm]	165 (150–184)	167 (155–179)	166.5 (150–184)	0.874
Weight [kg]	112 (80–171)	111 (85–150)	112 (80–171)	0.613
BMI [kg/m^2^]	40.6 (35.3–57.8)	41.2 (35.4–54.7)	40.8 (35.3–57.8)	0.553
Indication				0.459
Symptomatic cholecystolithiasis	33 (94.3)	29 (82.9)	62 (88.6)	
Cholecystitis	1 (2.9)	3 (8.6)	4 (5.7)	
Choledocholithiasis	1 (2.9)	2 (5.7)	3 (4.3)	
Biliary pancreatitis	0 (0)	1 (2.9)	1 (1.4)	
ASA				0.611
1	0 (0)	0 (0)	0 (0)	
2	13 (28.6)	10 (37.1)	23 (32.9)	
3	22 (71.4)	25 (62.9)	47 (67.1)	
History of major abdominal surgery				1.000
Yes	5 (14.3)	6 (17.1)	11 (15.7)	
No	30 (85.7)	29 (82.9)	59 (84.3)	
Preoperative pain [NRS]				0.741
0	31 (88.6)	31 (88.6)	62 (88.6)	
1	1 (2.9)	0 (0)	1 (1.4)	
2	1 (2.9)	2 (5.7)	3 (4.3)	
3	1 (2.9)	2 (5.7)	3 (4.3)	
7	1 (2.9)	0 (0)	1 (1.4)	
Need of preoperative analgesics				1.000
Yes	4 (11.4)	4 (11.4)	8 (11.4)	
No	31 (88.6)	31 (88.6)	62 (88.6)	

Values are reported as median (min–max) and counts (percentage)

*NC* transvaginal hybrid NOTES cholecystectomy, *LC* traditional 4-trocar laparoscopic cholecystectomy, *BMI* Body Mass Index, *NRS* Numeric Rating Scale, *ASA* American Society for Anesthesiologists

**Fig. 2:**
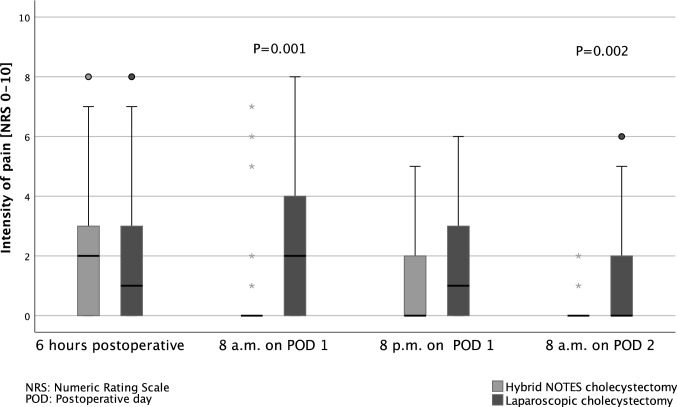
Daily intensity of pain

**Fig. 3:**
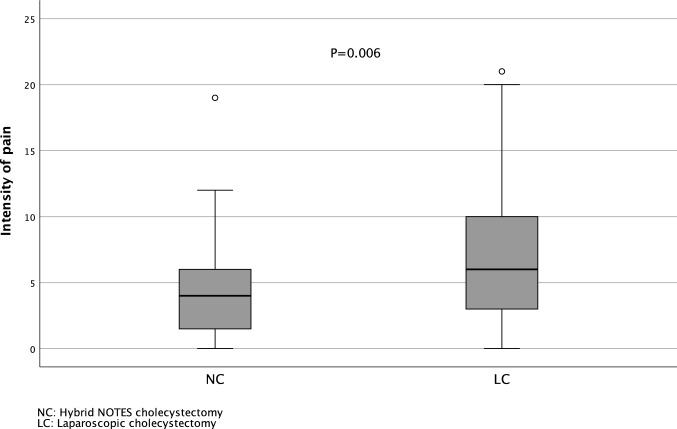
Cumulative pain

The postoperative pain on the morning of the first postoperative day (*P* = 0.001) and on the morning of the second postoperative day (*P* = 0.002) as well as the sum of pain measurements from the day of surgery to the morning of the second postoperative day (*P* = 0.006) was significantly lower in the NC group compared to the LC group (Fig. 2 and Fig. 3).

**Fig. 4:**
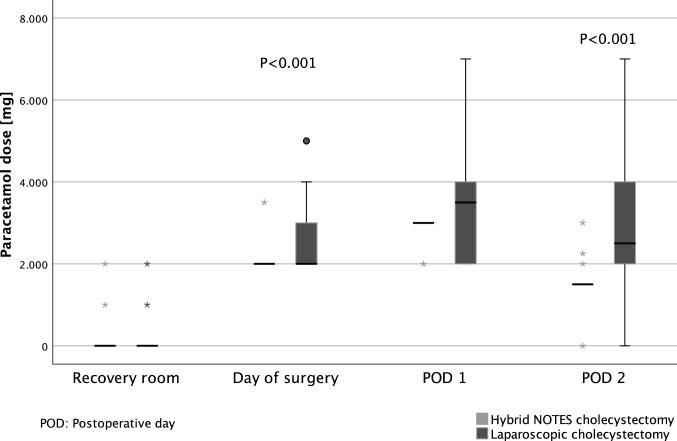
Daily use of paracetamol

**Fig. 5:**
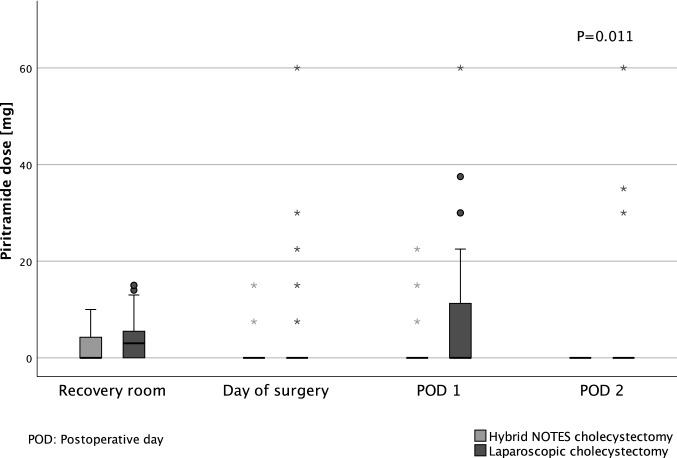
Daily use of piritramide

The intake of peripheral analgesics (paracetamol) was significantly lower in the NC group compared to the LC group on the day of surgery (*P* < 0.001) and on the second postoperative day (*P* < 0.001), whereas the differences in the recovery room and on the first postoperative day are not significant (Fig. 4). Figure 5 shows a significant reduction of the daily intake of centrally acting analgesics (piritramide) by the hybrid NOTES technique only at the second postoperative day (*P* = 0.011). Table 2 reflects the cumulative amounts of peripheral and centrally acting analgesics from surgery to the second postoperative day, which were both significantly lower in the NC group (*P* = 0.005 and *P* = 0.047).

**Table 2:** Patient outcome

Variable	NC (*n* = 35)	LC (*n* = 35)	Total (*n* = 70)	*P* value
Procedural time [min]	68 (31)*	70 (29)*	69 (29)*	0.643
Demand medication requested	9 (25.7)**	13 (37.1)**	22 (31.4)**	0.440
Cumulative paracetamol, from DOS to POD 2 [mg]	6,500 (0)*	9,000 (5,000)*	6,500 (3125)*	0.005
Cumulative piritramide, from DOS to POD 2 [mg]	1.5 (7.5)*	5.0 (27.0)*	4.5 (15.0)*	0.047
Postoperative complications	1 (2.9)**	5 (14.3)**	6 (8.6)**	0.198
Clavien–Dindo classification of postoperative complications				0.198
No complication	34 (97.1)**	30 (85.7)**	64 (91.4)**	
Grade I	1 (2.9)**	5 (14.3)**	6 (8.6)**	
Grades II to V	0 (0)**	0 (0)**	0 (0)**	
Postoperative hospital stay [days]	2 (0)*	3 (1)*	2 (1)*	< 0.001
Mortality	0**	0**	0**	

Values are reported as median (interquartile range)* and counts (percentage)**

*NC* transvaginal hybrid NOTES cholecystectomy, *LC* traditional 4-trocar laparoscopic cholecystectomy, *DOS* day of surgery, *POD* postoperative day

There were no significant differences between the groups in procedural time, demand of medication, postoperative complications and their classifications due to Clavien/Dindo, and mortality (Tab. 2). In contrast, the postoperative length of stay, also listed in Table 2, was significantly shorter in the NC group than in the LC group (*P* < 0.001).

In 21 patients, no additional transcutaneous auxiliary trocar had to be used. In 11 patients one additional trocar was necessary, in one patient two and in two cases even three auxiliary trocars were required.

## Discussion

Using a retrospective group comparison of 35 very obese patients (BMI ≥ 35 kg/m^2^) in each group who had undergone traditional laparoscopic cholecystectomy or transvaginal/transumbilical hybrid NOTES cholecystectomy, we found significant advantages for the hybrid NOTES group in terms of less pain despite lower use of peripheral and centrally acting analgesics, as well as a significantly shorter postoperative length of stay. Although the difference in cumulative piritramide dose showed only marginal significance, this may possibly be due to the high-dose variation.

Minimal invasive surgery (MIS) is designed to reduce access trauma during resection and reconstructive intra-abdominal procedures by replacing laparotomy with minimized trocar access with the goal of shortening postoperative hospital stay and reconvalescence by reducing pain, among other benefits. NOTES is the logical evolution of MIS, in which trocar access and retrieval incision through the abdominal wall are also eliminated and access to the abdominal cavity is achieved by penetrating an intra-abdominal hollow organ through natural body orifices, such as the stomach (transoral) or the posterior vaginal vault (transvaginal) [8, 9]. A great benefit of this approach is the lack of pain reception due to nonexistent pain fibers in the area of the posterior vaginal vault, for example, [10] and thus a complete reduction of pain resulting from the access path. However, this only partly accounts for intraoperative and postoperative pains; postoperative analgesia is often still necessary. The intra-abdominal surgery itself is not different from the laparoscopic technique. Thus, it only uses a different access pathway and is not a completely different procedure. Because pure NOTES procedures make triangulation difficult or would require the use of flexible instruments with inadequate intraperitoneal navigation, NOTES is usually supplemented in the routine clinical practice by one or more transcutaneous auxiliary trocars, which has led to the term "hybrid NOTES." The hybrid technique is the leading technique in most hospitals performing NOTES procedures [11].

Several meta-analyses have shown various advantages of transvaginal NOTES over traditional laparoscopy, such as less postoperative pain, less postoperative analgesic medication, a shorter length of stay, and a shorter time of recovery with no statistically different intra- and postoperative complication rates [2, 12, 13]. However, obese patients were mostly excluded in the underlying studies, as we already elaborated on in 2018 [4]. Obesity is a risk factor for the development of cholecystolithiasis and the need for surgical therapy [5, 14]. Furthermore, significant weight loss, which is more commonly sought in obese patients, whether achieved by diet or surgery, is also an independent risk factor for the development of gallstone disease [15, 16]. Thus, the question arises whether NC, which is beneficial for normal or overweight to mildly obese individuals, is also feasible and beneficial for the high-risk group of the very obese. After demonstrating in 2018 that NC is also feasible in very obese patients without increasing intraoperative or postoperative complication rates compared with NC in normal weight patients, as well as resulting in comparable postoperative lengths of stay [4], we sought to clarify whether the advantages comparing the two surgical techniques are also demonstrable in patients with a BMI of at least 35 kg/m^2^. Therefore, we performed a retrospective analysis of patients from the aforementioned study in terms of outcome parameters compared with LC patients of the same time period.

In obese women, the esthetic results are sometimes disputable, which is why we did not analyze this point.

A limit of our analysis is the retrospective study design, although we tried to minimize presumptive bias by selecting patients according to comparable patient-side parameters such as age and surgical urgency from the same time period. Despite some matching the percentage of uncomplicated cases (e.g., no history of inflammation) was lower in the traditional laparoscopic group compared to the hybrid which may represent some bias, although the difference was not significant. Nevertheless, this retrospective setting is a high limitation, and a prospective study in a multicenter setting is needed to confirm our findings. Another limitation of the study is the small number of patients.

Furthermore, NC is performed primarily in centers of excellence in minimally invasive surgery by very skilled surgeons, so the results should be applied with caution to daily practice.

## Conclusion

This is the first analysis comparing postoperative short-term parameters after NC versus LC in very obese patients (BMI ≥ 35 kg/m^2^). We found advantages of NC in terms of significantly less pain despite less analgesic use as well as significantly shorter postoperative length of stay for NC patients. Thus, we were able to confirm the advantages shown in normal weight to slightly obese patients for this group of patients, who are particularly at risk of gallstone disease and the need of cholecystectomy.
